# Therapeutic Development of Levosimendan in Acute and Advanced Heart Failure: A Systematic Review

**DOI:** 10.7759/cureus.37844

**Published:** 2023-04-19

**Authors:** Heet N Desai, Leslie Sangurima, Maujid Masood Malik, Nency Ganatra, Rosemary Siby, Sanjay Kumar, Sara Khan, Srilakshmi K Jayaprakasan, Doju Cheriachan, Lubna Mohammed

**Affiliations:** 1 Internal Medicine, California Institute of Behavioral Neurosciences and Psychology, Fairfield, USA; 2 Biomedical Sciences, King Faisal University, Alhsa, SAU; 3 Biomedical Sciences, California Institute of Behavioral Neurosciences and Psychology, Fairfield, USA; 4 Internal Medicine, Bahria University Medical and Dental College PNS Shifa Hospital, Karachi, PAK; 5 Pediatrics, Dr. B.R. Ambedkar Medical College and Hospital, Bengaluru, IND; 6 Pediatrics, California Institute of Behavioral Neurosciences and Psychology, Fairfield, USA; 7 Emergency Medicine, Stepping Hill Hospital, Stockport, GBR; 8 Emergency Medicine, California Institute of Behavioral Neurosciences and Psychology, Fairfield, USA

**Keywords:** calcium sensitizing inotrope, levosimendan, advanced cardiac failure, decompensated cardiac failure, acute cardiac failure, congestive heart failure, right ventricular failure, left ventricular failure, cardiac failure, heart failure

## Abstract

Levosimendan (LS) has been progressively used for the treatment of patients developing acute as well as chronic or advanced cardiac dysfunction. It has proven to be a better inotropic agent than its counterparts in terms of its ability to increase the cardiac output in an acutely or chronically decompensated heart without an increase in the myocardial oxygen demand. The purpose of this systematic review, which was carried out in accordance with Preferred Reporting Items for Systematic Reviews and Meta-analyses (PRISMA) 2020, was to determine the efficacy and advantages of utilizing LS in patients with both acute and chronic heart failure. We collected and reviewed articles, including clinical trials, literature reviews, randomized and non-randomized control trials, case-control and cohort studies, and systematic reviews and meta-analyses published between January 1, 2012, and November 27, 2022. The databases that were used to collect these articles included Pubmed, Pubmed Central, Cochrane Library, and Google Scholar. After applying appropriate filters, a total of 143 reports were identified from these four databases. They were further screened and subjected to quality assessment tools which finally yielded 21 studies that were included in this systematic review. This review provides strong evidence that the pharmacological properties and different mechanisms of action of LS give it an upper hand over other inotropic agents for its successful administration in patients with either acute or advanced cardiac failure, which consists of left as well as right ventricular failure, either individually or in combination.

## Introduction and background

Heart failure (HF) is a long-term clinical condition that can be brought on by problems with the heart valves, pericardium, myocardium, or endocardium. It can also happen as a result of certain metabolic imbalances [1]. Patients with severe symptoms, frequent de-compensation, and clearly noticeable significant cardiac dysfunction are referred to as having "advanced HF (AdHF)" [1], whereas acute HF (AHF) is characterized by the sudden development of decompensated symptoms and/or HF indicators and is a potentially fatal condition with a short-term mortality rate of about 30% [1-3]. Breathing difficulties, orthopnea, leg edema, and weariness are typical symptoms of HF [4]. Because of frequent hospitalizations owing to acute de-compensation or aggravation, HF reduces the quality of life by increasing morbidity and mortality. Close to 1-2% of the adult population has HF, with a frequency of more than 10% in those over 70, and death from acute de-compensation may be as high as 10% at 60 days, with a 50% likelihood of new hospitalizations in the subsequent six months [3].

One of the main causes of AHF is acute coronary syndrome (ACS), which typically occurs in conjunction with severe myocardial dysfunction and damage as well as arrhythmia [5]. However, AHF can also develop without any known precipitating factors as a result of a primary cardiac dysfunction caused by myocardial, endocardial, or heart valve defects, as well as from a variety of other causes, including poor dietary choices, noncompliance with treatment instructions, infections, or metabolic abnormalities [1]. Extremely quick mortality follows AHF brought on by either an ACS episode or an infection [6].

There is a greater need for new therapeutic approaches that can slow or prevent clinical progression and prevent hospitalization outside of the drug and device therapy recommended by guidelines because many patients with AHF and chronic HF have few specific therapeutic options, and these options frequently fail to slow disease progression [7]. Patients with AdHF may benefit from occasional intervals of hemodynamic respite from inotropic medication infusions, which have been linked to improvements in symptoms [7]. Unfortunately, there is no treatment plan for acutely decompensated HF that is effective in all cases with various etiologies. For example, in patients with known HF, the use of beta agonists may increase all-cause mortality, HF hospitalization, and the use of vasodilators, whereas the use of vasopressors increases vasoconstriction, increasing systemic vascular resistance, and deteriorating acute decompensated HF [4]. Due to its unique mode of action, levosimendan (LS), a calcium sensitizer and potassium channel opener, has recently drawn interest as a potential replacement for inotropy that may be safer than the conventional classes of cardio-mobilizing medications [8].

The early 1990s saw the development of LS in Finland, and it was only in 2001 that it was made prescription-only [9]. It has been employed in 60 countries since then [9]. LS is an inodilator used to treat decompensated HF, and its pharmacological effects are exerted through three different mechanisms: (1) increased calcium sensitivity of troponin C in myocardial cells, leading to a cAMP-independent inotropic effect; (2) opening of potassium channels (K-ATP channels) that are adenosine triphosphate-sensitive and cause vasodilation in smooth muscle cells of the vasculature; and (3) activation of K-ATP channels in cardiac mitochondria, preventing cells from suffering damage from ischemia/reperfusion [7,10-17]. The effects of LS include an improvement in cardiac contractility, an increase in cardiac output and stroke volume, a drop in pulmonary artery pressure and pulmonary capillary wedge pressure (PCWP), and a reduction in systemic and vascular resistance [2,11]. Various molecular actions and their respective pharmaceutical results of LS have been elaborated in Table 1 [8].

**Table 1:** Molecular targets and pharmaceutical results of LS ATP: adenosine triphosphate Cardiovascular and organ (heart) protection: Anti-ischemic effect due to the blocking of the potassium channels by ATP in the inner mitochondrial membrane Modified from Agostoni et al. [8]

Molecular Targets	Pharmaceutical Results
By specifically binding to calcium-saturated cardiac troponin C, calcium sensitization of the contractile machinery is achieved	Inotropy with no rise in calcium transient or oxygen consumption; anti-stunning impact
ATP-sensitive channels on vasculature’s smooth muscle open	Vasodilation (includes coronary arteries as well); elevated end-organ perfusion
Activation of the ATP-sensitive potassium channels in the mitochondria	Cardiovascular and organ (heart) protection; ischemic impact prevention

The pharmacokinetics of LS is another factor that sets it apart, especially the way that a long-acting active metabolite known as OR-1896 forms in the intestines through a reduced acetylation pathway [6,11]. While having a significantly longer plasma half-life than the parent drug, this molecule shares the same pharmacologic and hemodynamic characteristics as the latter, helping the latter's therapeutic benefits to last longer after only one infusion [6].

LS has been researched for potential application in a range of therapeutic contexts, such as the management of AHF, patients with low cardiac output, and high-risk cardiac surgery. Along with right ventricular failure, cardiogenic shock (CS), septic shock, and takotsubo cardiomyopathy, it has also shown early encouraging outcomes in a number of other disorders requiring inotropic support [10,17]. ​There have been various articles and publications explaining different aspects of LS in terms of its use in varied HF stages over the past 10 years. This systematic review aggregates important information about the pharmacokinetics and therapeutic development of LS in the last decade in patients with acute and chronic HF, including left as well as right cardiac dysfunction.

## Review

Methods

LS use in patients with acute and/or severe cardiac failure is the subject of this systematic review, which aims to investigate and evaluate it. This systematic review was carried out in accordance with the Preferred Reporting Items for Systematic Reviews and Meta-Analyses (PRISMA) 2020 recommendations.

Search Strategy

A thorough literature search of databases such as Pubmed, Pubmed Central (PMC), Cochrane Library, and Google Scholar was done. The search was generated using keywords such as "heart failure," "cardiac failure," "left ventricular failure," "right ventricular failure," "congestive heart failure," "left ventricular systolic dysfunction," "acute cardiac failure," "decompensated cardiac failure," "advanced cardiac failure," "postural nocturnal dyspnea," "dyspnea on exertion," "orthopnea," "Levosimendan," "inodilator," and "calcium sensitizing inotrope" and combining them using the BOOLEANs “AND” and “OR.” A mesh strategy was used to narrow down the published articles. Table 2 summarizes the databases screened for collections of articles and the search strategy used for the same.

**Table 2:** Databases used for collecting articles (along with search strategies and appropriate filters) PMC: Pubmed Central

Type of database	Keywords	Search strategy	Filters used	No. of records
Pubmed	Heart failure, Cardiac failure, Left ventricular failure, Right ventricular failure, Congestive heart failure, Left Ventricular Systolic Dysfunction, Acute Cardiac failure, Decompensated cardiac failure, advanced cardiac failure, Postural Nocturnal Dyspnea, Dyspnea on exertion, Orthopnea, Levosimendan, Inodilator, Calcium sensitizing inotrope	Heart failure OR Cardiac failure OR Left ventricular failure OR Right ventricular failure OR Congestive heart failure OR Left Ventricular Systolic Dysfunction OR Acute cardiac failure OR Decompensated cardiac failure OR Advanced cardiac failure OR Postural Nocturnal Dyspnea OR Dyspnea on exertion OR Orthopnea OR (("Heart Failure, Systolic"[Mesh] AND "Heart Failure, Diastolic"[Mesh] AND "Heart Failure"[Mesh]) AND ("Heart Failure/analysis"[Mesh] OR "Heart Failure/classification"[Mesh] OR "Heart Failure/complications"[Mesh] OR "Heart Failure/diagnosis"[Mesh] OR "Heart Failure/diagnostic imaging"[Mesh] OR "Heart Failure/genetics"[Mesh] OR "Heart Failure/physiology"[Mesh] OR "Heart Failure/physiopathology"[Mesh] OR "Heart Failure/prevention and control"[Mesh] OR "Heart Failure/therapy"[Mesh])) AND ("Heart Failure/mortality"[Mesh] OR "Heart Failure/statistics and numerical data"[Mesh] OR) AND Levosimendan OR Inodilator OR Calcium sensitizing inotrope OR ("Simendan/administration and dosage"[Mesh] OR "Simendan/adverse effects"[Mesh] OR "Simendan/analysis"[Mesh] OR "Simendan/chemistry"[Mesh] OR "Simendan/economics"[Mesh] OR "Simendan/metabolism"[Mesh] OR "Simendan/organization and administration"[Mesh] OR "Simendan/pharmacokinetics"[Mesh] OR "Simendan/pharmacology"[Mesh] OR "Simendan/physiology"[Mesh] OR "Simendan/standards"[Mesh] OR "Simendan/therapeutic use"[Mesh])	Free full text, Books and Documents, Clinical Trial, Meta-Analysis, Randomized Controlled Trial, Review, Systematic Review, in the last 10 years, Humans, Middle Aged + Aged: 45+ years	24
PMC	levosimendan, calcium sensitizing inotrope, heart failure	levosimendan AND calcium sensitizing inotrope AND heart failure	Published in the last 10 years	79
Cochrane Library	Levosimendan, Heart failure	Levosimendan AND Heart failure	Published in the last 10 years	01
Google Scholar	Levosimendan, Acute heart failure, Decompensated heart failure	allintitle: Levosimendan AND Acute heart failure OR Decompensated heart failure	Published in the last 10 years	39

Eligibility Requirements

Based on the following participant, intervention, and outcome characteristics, the studies were chosen for inclusion:

Participants: Adult and geriatric populations (>= 18 years) of all ethnicities and genders with signs of early, AHF, and AdHF were chosen for studies.

Intervention: Use of LS in the abovementioned population.

Outcomes: LS administration appears to be effective in symptomatically improving or halting the progression of de-compensation in patients with HF, according to studies.

Inclusion and Exclusion Criteria

Additionally, the following inclusion and exclusion standards were added: papers were only taken into consideration if they were written entirely in English, free full-text articles published within the previous 10 years, randomized control trials (RCTs), non-RCTs, case series, and case reports; cohort studies; case-control studies; systematic reviews; literature reviews; and meta-analyses. Animal studies weren’t considered for this systematic review.

Results

**Figure 1:**
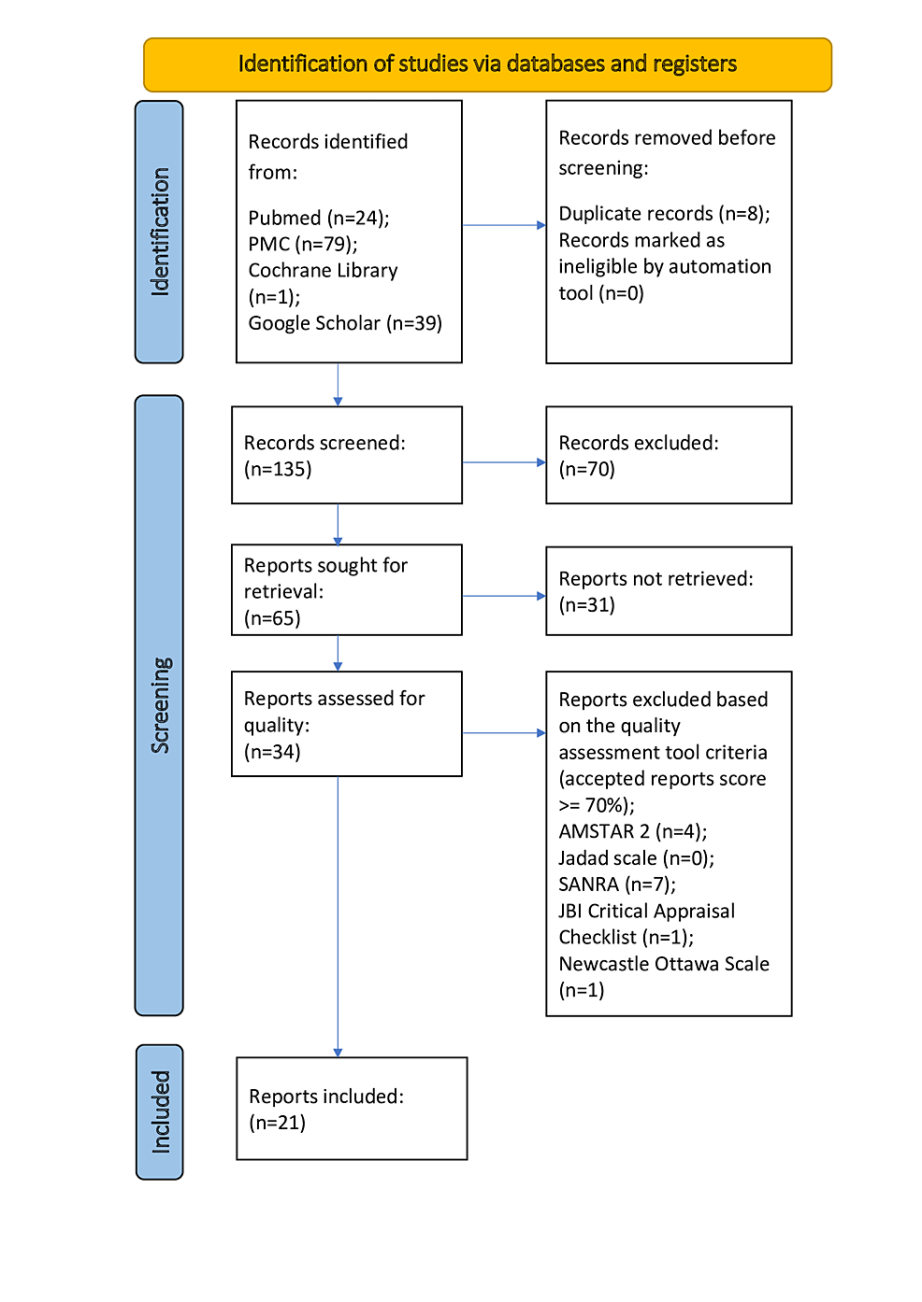
Screening process and quality assessment of the articles (PRISMA chart) PMC: Pubmed Central AMSTAR 2: Assessment of Multiple Systematic Reviews 2 SANRA: Scale for the Quality Assessment of Narrative Review Articles JBI Critical Appraisal Checklist: Joanna Briggs Institute Critical Appraisal Checklist PRISMA: Preferred Reporting Items for Systematic Reviews and Meta-Analyses

Using the search strategies and appropriate filters, a total of 143 articles were identified from the databases, as mentioned above, within the last 10 years (January 1, 2012, to November 27, 2022). The screened articles, after the removal of duplicates and irrelevant records, were subjected to quality assessment by using quality assessment tools such as AMSTAR 2 (for systematic reviews and meta-analyses), the Jadad scale (for RCTs and non-RCTs), SANRA (for narrative review articles), the JBI quality appraisal checklist (for case series and case reports), and the Newcastle-Ottawa checklist (for case-control and cohort studies). An overview of the screening process is presented in the PRISMA chart, as shown in Figure 1.

The studies included in this systematic review are summarized in Table 3.

**Table 3:** Included studies SANRA: Scale for the Quality Assessment of Narrative Review Articles AMSTAR 2: Assessment of Multiple Systematic Reviews 2

Author	Publication year	Report type	Quality assessment tool used	Score
Nieminen et al. [10]	2013	Review article	SANRA*	10
Agostoni et al. [8]	2019	Review article	SANRA	11
Herpain et al. [17]	2018	Review article	SANRA	11
Lee et al. [4]	2022	Cohort study	Newcastle Ottawa scale#	8
Gracia-Gonzalez et al. [15]	2021	Randomized control trial	Jadad scale^	7
Ortis et al. [13]	2017	Case-control study	Newcastle Ottawa scale	7
Pashlovetsky et al. [1]	2019	Review article	SANRA	11
Harjola et al. [6]	2018	Literature review	SANRA	11
Cholley et al. [9]	2019	Review article	SANRA	10
Uhlig et al. [18]	2020	Systematic review	AMSTAR 2**	13
Hu et al. [19]	2021	Systematic review and meta-analysis	AMSTAR 2	14
Najjar et al. [20]	2018	Cohort study	Newcastle Ottawa scale	7
Comin-Colet et al. [7]	2018	Randomized control trial	Jadad scale	7
Cornejo-Avendano et al. [3]	2017	Systematic review and meta-analysis	AMSTAR 2	15
Tasal et al. [2]	2014	Cohort study	Newcastle Ottawa scale	9
Bouchez et al. [16]	2018	Review article	SANRA	11
Morelli et al. [21]	2014	Review article	SANRA	10
Farmakis et al. [14]	2016	Review article	SANRA	11
Altenberger et al. [11]	2017	Literature review	SANRA	11
Wang et al [12]	2019	Randomized control trial	Jaded scale	7
Nieminen et al. [5]	2016	Literature review	SANRA	12
*SANRA checklist accepted score (>=70%): Minimum score 9 out of 12; #Newcastle Ottawa scale accepted score (>=70%): Minimum score 6 out of 9; ^Jadad scale accepted score (>=70%): Minimum score 6 out of 8; **AMSTAR 2 checklist accepted score (>=70%): Minimum score 12 out of 16				

Discussion

**Figure 2:**
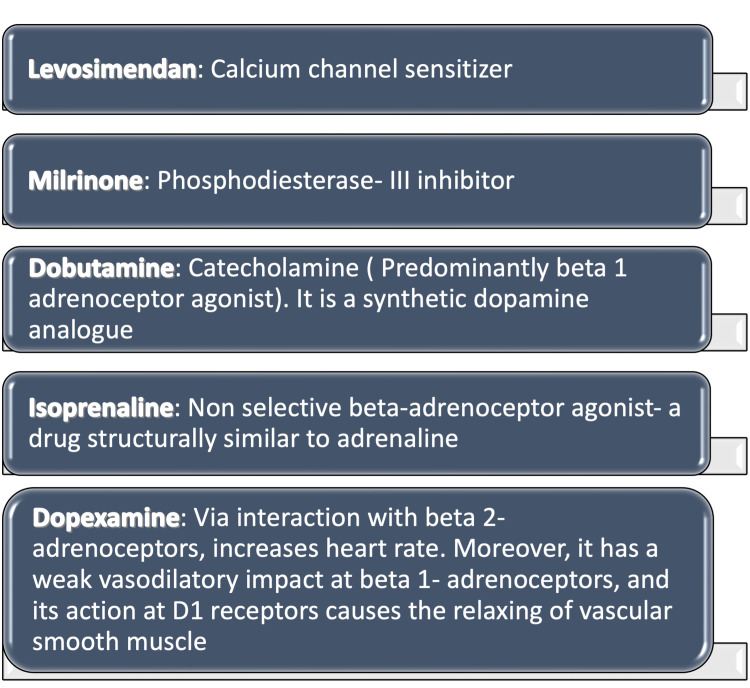
Mechanisms of action of various positive inotropic agents Other examples of catecholamine are epinephrine; norepinephrine D1 receptors: dopamine 1 receptors

When conventional therapy was deemed insufficient and inotropic support was deemed appropriate, LS was initially approved in Sweden in 2000 for the short-term treatment of severely decompensated chronic HF [20,22]. The mechanisms of action of some of the positive inotropic agents are expanded in Figure 2.

**Figure 3:**
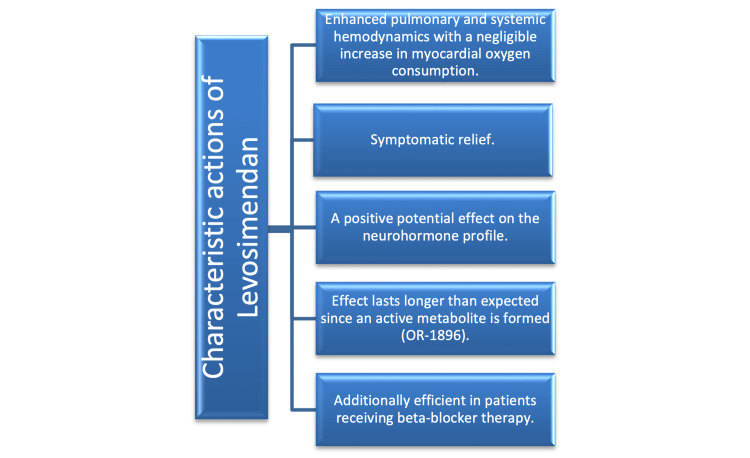
LS characteristic behaviors OR-1896: A phosphodiesterase III isoform inhibitor and a vasodilator Modified from Bouchez et al. [16]

The current indication for LS as a short-term treatment for acutely decompensated severe chronic HF is supported by clinical trial data, from which a number of noteworthy characteristics were extracted and explained diagrammatically in Figure 3 [16].

Pharmacokinetics of LS

**Figure 4:**
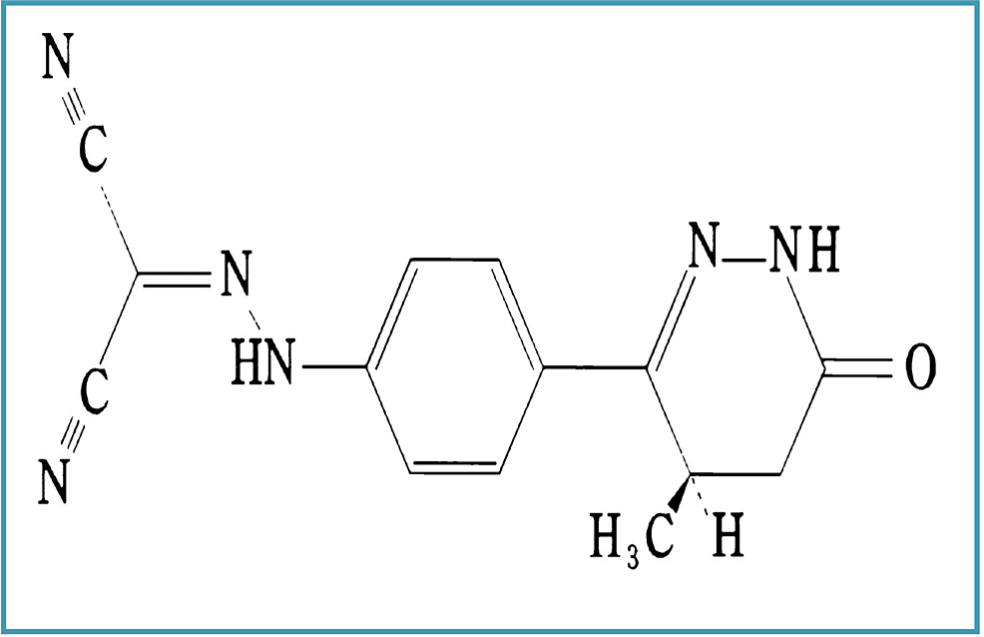
Chemical composition of LS

With a pKa of 6.2 and a half-life of around one hour, LS is a weak acid and a moderately lipophilic molecule [23]. The chemical name for LS is (-) (R)- [4-(1,4,5,6-tetrahydro-4-methyl-6-oxo-3-pyridazinyl) phenyl] hydrazono] propanedinitrile, according to WHO Drug Information Vol. 7, No. 3, 1993. Its molecular weight is 280.291, and its chemical formula is C14H12N6O, which is depicted in Figure 4 [23].

The majority of the drug's metabolism occurs at one of its nitrile groups via glutathione conjugation, followed by amino acid cleavage and cyclization or acetylation [9,23]​. However, a tiny amount of the medication (5% of the dose) is transformed into a metabolite, OR-1855, during the metabolism of LS in the large intestine and subsequently into the active metabolite, OR-1896, in the liver by a reduction-acetylation route [1,22,23]​. The cardiovascular therapeutic effects of the metabolite OR-1896 last for several days even after the parent drug's 24-hour infusion has been stopped, due to the metabolite’s significantly elevated elimination half-life of 80 hours as compared to that of the parent drug of one hour, and it has hemodynamic and pharmacologic effects that are comparable to those of LS [1,10,22,24,25]​. All these traits of LS allow it to deliver the best therapeutic response to certain circumstances [9].

Typically, the infusion is started at 0.1 g/kg/min and raised to 0.2 g/kg/min for 24 hours as long as the systolic blood pressure (SBP) remains constant for the first two to three hours. A 24-hour break between infusions is advised for patients with AHF [1]. The infusion rate can be lowered to 0.05 µg/kg/min or stopped if symptomatic hypotension or tachycardia develops [15]. To reduce the risk of hypotension in patients with systolic or diastolic blood pressure less than 100 mm Hg or 60 mm Hg, respectively, an initial bolus of LS is typically not administered [1,9,15]​.

LS in AHF

AHF is a circumstance in which the signs and symptoms of HF suddenly appear or get worse, and it must be classified as a life-threatening medical illness that necessitates immediate assessment and treatment and frequently ends with the patient getting hospitalized [8]. Finding individuals with CS and/or respiratory failure, which account for about 10% of critically ill patients requiring intensive care, is a top priority while working up a case of probable AHF [8]. A fundamental pathophysiological feature of HF is that it flattens the increase in cardiac output at a given afterload, resulting in "forward" failure [8]. The use of inotropic medications can be a viable approach to this scenario, although the available medicines are limited [8].

Traditional adrenergic inotropes and phosphodiesterase (PDE)-3 inhibitors are sometimes linked to increased mortality, arrhythmias, or other safety problems, according to a number of clinical trials and meta-analyses undertaken over the past 25 years [8,17]​. In contrast, LS, which has been in use for over 20 years and has been tested in controlled clinical trials involving over 3000 HF patients, represents an established inotropic therapy in AHF because it does not alter cellular oxygen demand or calcium content, therefore having a better safety profile [8]. LS enhances inotropic action without increasing myocardial oxygen consumption due to its specific binding to troponin C in the myocardial cells [25]. According to the findings of a meta-analysis combining data from over 6000 patients and a real-world registry encompassing over 5000 patients [25], LS may improve long-term survival, at least relatively long-term survival. LS may be considered one of the few inotropes, and in some cases, the only one for which a strong case can be made for its use [8].

In patients with obvious signs of pulmonary edema and excessive PCWP who do not respond appropriately to vasodilation, LS is recognized for its greater ability to decrease PCWP and right atrial pressure compared to other inotropes like dobutamine [6,10,26]. According to the Randomized Multicenter Evaluation of Intravenous Levosimendan Efficacy Trial II (REVIVE II trial; n = 600), which randomly assigned patients in a double-blind study, intravenous LS was given to individuals with acute decompensated HF, a mean left ventricular ejection fraction of less than or equal to 23%, dyspnea at rest, and who were receiving diuretics [1,27]. The primary goal of change in the clinical course over five days demonstrated greater gains (by 33%) in the LS group when compared to the placebo group [1,27].​

The key to resolving the issue of AHF lies in understanding the pathophysiology that causes it. Because AHF results from fluid redistribution, increased left ventricular pressure, and vasoconstriction with increased venous return, it is a phenotype that responds well to therapy with vasodilators [8]. AHF caused by poor cardiac output is the correct target for inotrope therapy [8]. Several findings demonstrate the need for better methods to identify people who truly require inotropic care [8]. Patients who are already on beta-blockers, those with ischemic AHF, and those with cardio-renal syndrome should always receive LS as their first choice of treatment among those who meet the criteria [8]. In individuals with AHF syndromes, LS is the only medication that so far appears to induce clinical improvement that lasts past the course of treatment [22].

LS in AHF or CS Secondary to ACS

AHF in the setting of ACS is an urgent issue that necessitates quick detection and care, not the least of which is the possibility that AHF will progress into CS. Age, a previous myocardial infarction (MI), or chronic HF are risk factors for the development of AHF in ACS, as are diabetes, hypertension, and the female sex [8]. AHF is still a significant consequence of ACS, affecting a considerable portion of patients, and is connected to worse outcomes, even if its prevalence as an ACS complication has decreased recently [6]. While the classic hemodynamic profile of CS includes low cardiac output, low arterial pressure, elevated left/right ventricular diastolic pressure, and elevated systemic vascular resistance, other phenotypes can occur, such as ischemic-reperfusion injury states that result in a sepsis-like syndrome with low systemic vascular resistance, while others present with maintained arterial pressure and signs of end-organ hypo-perfusion as a result. Primary percutaneous coronary intervention for ST-elevated MI, hydration treatment, vasopressors, and inotropes are the norm in CS. There is still a dearth of information on the application of LS in CS. Nevertheless, the medication seems to be secure and to enhance a few hemodynamic and ventricular indices [10].

The safety of LS was examined in the Randomized Study on the Safety and Effectiveness of Levosimendan in Patients with Left Ventricular Failure after an Acute Myocardial Infarction (RUSSLAN), a placebo-controlled, double-blind, parallel-group, randomized study with 504 patients enrolled in five days after an index infarction [10,28]. In this study, mortality was prospectively monitored for 14 days after treatment began which showed that LS significantly decreased mortality as compared to placebo (12% vs. 20%; p = 0.031), and this beneficial impact often lasted 180 days (23% vs. 31%; p = 0.053) [28]. With routine cardiac care and dobutamine, it is estimated that 288 out of 1000 patients with CS will pass away within a short period of time, compared to 89 (95% CI 53 to 152) patients with LS [18]. However, there is little difference between the two in the long term [18].

According to current expert advice on the use of LS in AHF complicating ACS, it can be thought about as a potential substitute for adrenergic medications in all patients who have had chronic beta-blocker therapy or in those whose urine production is insufficient after taking diuretics [6]. In addition, it might be appropriate for those with SBP readings between 85 and 100 mm Hg (corresponding to Killip Class III with acute pulmonary edema), and it might be the preferred treatment for those with CS when used in conjunction with noradrenaline or another vasopressor (roughly equivalent to Killip Class IV and SBP 85 mm Hg with indications of peripheral vasoconstriction) [5,6].​

LS in AHF Secondary to Septic Shock

Modern definitions of sepsis include an infection that causes at least one organ to malfunction due to an uncontrolled host inflammatory response. Septic cardiomyopathy (SCM), which results in de novo AHF as a result of cardiac depression, can also arise from sepsis in addition to the intrinsic distributive shock brought on by vascular hypo-reactivity and autonomic dysfunction [17]. Myocardial dysfunction can lead to a reduction in cardiac output, which may further lead to organ hypo perfusion, necessitating fast and effective therapy to re-establish cardiovascular function and reverse shock [21]. The large variation in SCM prevalence among septic patients (20% to 60%) reflects both the current inadequate classification and the multiplicity of the symptoms [17]. One of the primary characteristics of the early stage of SS is the presence of intense sympathetic activity, which causes tachycardia, vasoconstriction, and increased inotropism as a result of the body's natural attempt to maintain the perfusion of vital organs [21]. The septic heart has decreased contractility despite increased sympathetic outflow because of attenuation of the adrenergic response at the cardiomyocyte level, which is primarily mediated by cytokines and nitric oxide [21]. This results in a down-regulation of beta-adrenergic receptors and a depression of post-receptor signaling pathways [10,21]. This is a significant mechanism of sepsis-induced cardiac depression, as 75-80% of myocardial adrenergic receptors are beta-1 receptors [21]. Other factors that have been shown to contribute to septic myocardial dysfunction include suppressed L-type calcium currents, decreased ryanodine receptor density and activity, and changes in calcium re-uptake in the sarcoplasmic reticulum [21].

**Figure 5:**
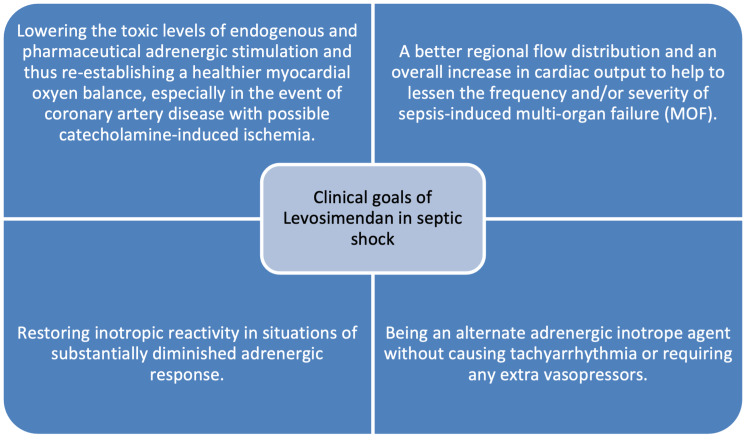
Therapeutic objectives of LS in septic shock MOF: multi-organ failure Modified from Herpain et al. [17]

For the recovery of a sufficient cardiac output and the delivery of peripheral oxygen, inotropic support is recommended [17]. Despite being regarded as a first-line inotrope medication in patients with reduced cardiac output brought on by septic shock, dobutamine's effectiveness has not been thoroughly established, and its effect on patients' survival is minimal [21]. On the other hand, taking into consideration the pathology explained in the previous paragraph, the benefits of administering LS in patients with septic hearts lie in the compound’s ability to enhance the inotropic effect without interacting with beta-adrenergic receptors and cAMP production, thus leaving the intracellular calcium concentration undisturbed [21]. Certain therapeutic objectives and criteria should be addressed by LS as an alternative therapy in a patient with SCM following septic shock, as indicated in Figure 5 [17].

A 24-hour infusion of either LS (0.2 g/kg/min) or dobutamine (5 g/kg/min) was randomly assigned to 28 septic patients with chronic left ventricular dysfunction following 48 hours of standard treatment in a trial to determine the effectiveness of LS in treating septic myocardial depression [29]. When compared to dobutamine, the use of LS was linked to increased cardiac output and pulmonary decongestion without a rise in the need for vasopressors, and a more favorable evolution of different multi-organ failure (MOF) surrogates like lactate clearance, veno-arterial carbon dioxide gap, gut mucosal perfusion, and renal perfusion [17,29]​. According to the currently known clinical evidence in septic shock, LS can successfully replace dobutamine in the treatment of de novo AHF caused by SCM, with additional beneficial extra cardiac effects due to the improvement of MOF [17].

LS in Chronic or AdHF

Hospitalizations for refractory HF patients are common because of clinical worsening [10]. Acute-on-chronic hemodynamic status deterioration, which may include situations where AHF is superimposed on AdHF, accounts for up to 80% of all hospitalizations [8]. During such admissions, individuals frequently get injected with vasodilators and positive inotropes like milrinone, dopamine, or dobutamine attempting to enhance heart function, assist diuresis, and support clinical stability [9,10]​. These individuals have a high mortality risk and may be waiting for a heart transplant or a long-term mechanical assist device, or alternatively, may be qualified for these therapeutic options [9]. Yet, despite encouraging hemodynamic and symptomatic improvements in small-scale clinical research, concerns have been raised concerning the safety of intermittent or continuous inotropic treatment [10].

The presently acknowledged mechanism for the onset and progression of HF is cardiac remodeling [2]. Pro-inflammatory markers, such as TNF-α, IL-6, and IL-1 levels in the bloodstream, have been linked to cardiac myocyte and endothelial cell death, decreased myocardial contractility, and clinical and hemodynamic HF worsening, and it is a key biological process linked to excessive neurohormonal stimulation that promotes left ventricular remodeling [2]. When treating decompensated chronic HF, changes in this neurohormone's (BNP) levels were closely linked to early re-hospitalization and mortality [2].

Evidence suggests that people with AdHF will live longer and require fewer hospital stays with intermittent use of LS [1]. According to several studies, LS helps these patients' clinical conditions by causing a notable drop in BNP and pro-inflammatory marker levels [2]. A multi-center, double-blind, randomized, parallel-group, placebo-controlled experiment called LION-HEART examined the effectiveness and safety of giving intermittent intravenous doses of LS to outpatients with AdHF [7]. A total of 69 patients were randomly assigned at a 2:1 ratio to receive LS (n = 48) or a placebo (n = 21) treatment via a six-hour intravenous infusion (0.2 g/kg/min without bolus) every two weeks for 12 weeks [7]. Furthermore, the LAICA trial which investigated the effectiveness and safety of long-term intermittent LS infusion was also a multicenter, prospective, randomized, double-blind, and placebo-controlled trial [15]. A total of 97 patients with AdHF who had experienced at least one acute decompensation or worsening HF hospitalization within the previous six months were randomly allocated to receive either LS (n = 70) or a placebo (n = 27) along with best practice standard HF treatment [15]. Both these investigations showed that patients in the LS arm of the research had lower NT-pro-BNP levels, fewer HF hospitalizations, and fewer HF-related deaths [1,7,15].

Left ventricular dysfunction is the most frequent cause of right ventricular failure, and bi-ventricular dysfunction has a poorer prognosis than pure left ventricular dysfunction [10]. Right ventricular failure is often the main cause of death in people with heart malfunctions, including those with systemic illnesses [19]. Low left ventricular filling pressure, elevated jugular venous pressure, low left ventricular filling pressure, and low output syndrome are all symptoms of isolated right ventricular failure [10]. The pathogenic elements of the primary disease should be the focus of the right HF treatment [19]. Patients with right ventricular failure have participated in investigator-initiated research studies, and it has been demonstrated that LS decreases the elevated right ventricular after-load, enhances right ventricular contractility, and enhances right ventricle diastolic performance [​10,19]​.

Limitations

This systematic review encounters certain limitations. As mentioned before, LS has been in clinical use since 2000, but the majority of the information gathered in this review has been included in the articles, studies, and clinical trials that have been published in the last 10 years. In addition, only the free full-text articles available across various databases have been used for this review. Publications in languages other than English were rejected during the screening process. Studies with minors (i.e., age below 18 years), including the pediatric population, as the subjects, were not included in this review; thus, the congenital heart conditions leading to HF have not been discussed in this systematic review. Studies in which animal models have been used to test the efficacy and safety of LS have been excluded from the review. Drug interactions and side effects of LS have not been explained in this review. Lastly, the use of LS during cardiac surgeries has not been taken into consideration while describing the pharmaceutical benefits of the drug.

## Conclusions

This systematic review proves the efficacy of LS in patients with AHF and AdHF. Although LS itself has a very short half-life, its active metabolite, which has similar pharmaceutical properties as that of the original drug, has a much longer half-life and is hence primarily responsible for carrying out the actions of LS. Various mechanisms of action of this drug include a positive inotropic effect independent of cAMP activity, a vasodilatory effect owing to the opening of the K-ATP channels in the smooth muscles, and the prevention of ischemic or re-perfusion myocardial injury by its action on K-ATP channels in the mitochondria of the cardiac muscles. It is by virtue of these properties that LS has been increasingly used in patients with AHF, both de novo and secondary to other conditions, and for the treatment of chronic or AdHF, as it has proved to be a better option for these patients compared to other inotropes such as dobutamine. This review has very clearly marked and explained the use of LS in both acute and chronic cardiac failure and gathered enough evidence to support this statement from various clinical studies and trials performed in the last decade. Suggestions for future research include clinical trials to study interactions of LS with other classes of drugs, such as antibiotics, anti-psychotics, anesthetics, and anti-arrhythmic agents, clinical trials for using LS in the pediatric population with congenital heart conditions leading to HF in adult life.
